# Ventricular Arrhythmias Caused by Left Main Coronary Artery Vasospasm: A Diagnostic Challenge in a Cardiac Arrest Victim

**DOI:** 10.7759/cureus.79554

**Published:** 2025-02-24

**Authors:** Inês Carqueja, Fernando Montenegro Sá, Sofia Monteiro

**Affiliations:** 1 Intensive Care Unit, Hospital Pedro Hispano, Matosinhos, PRT; 2 Cardiology Service, Department of Medicine, Hospital Pedro Hispano, Matosinhos, PRT

**Keywords:** cardiac dysrhythmia, coronary artery vasospasm, out-of-hospital cardiac arrest, post-resuscitation care, sudden cardiac death (scd)

## Abstract

Sudden cardiac death (SCD) is a common cause of cardiovascular deaths. It may be caused by primary electrical diseases, cardiomyopathies, myocarditis, valvular heart diseases, or coronary artery disease (including acute coronary syndrome).

Coronary artery vasospasm is defined as a transient total or subtotal coronary artery obstruction, associated with angina symptoms and ischemic findings on electrocardiogram (ECG). It is a cause of myocardial infarction, life-threatening arrhythmias, atrioventricular block, and SCD.

A 55-year-old man presented to the hospital after an out-of-hospital cardiac arrest (OHCA). He had a previous history of cardiovascular risk factors, excessive alcohol intake, non-obstructive coronary artery disease, and paroxysmic atrial fibrillation.

On the day of the OHCA, he had a sudden collapse while exercising, with ventricular fibrillation and return of spontaneous circulation (ROSC) after advanced life support (ALS). No ECG or echocardiographic anomalies were identified on hospital admission. The patient suffered three more episodes of cardiac arrest during the hospital stay, with atypical arrhythmic presentations. No ECG or echocardiographic abnormalities were observed after ROSC. The fourth cardiac arrest had concomitant segmental ST changes and de novoechocardiographic segmental motility abnormalities suggestive of left main coronary artery occlusion. These findings were transient, with a normal ECG and echocardiogram obtained one hour after ROSC. No electrolyte abnormalities or other causes of cardiac arrest were identified. The hypothesis of left main coronary vasospasm was raised as the likely diagnosis.

Coronary artery vasospasm is a possible cause of major cardiac events. Diagnosis can be challenging due to the transient findings and varied manifestations, often in patients with normal coronary arteries. The correct diagnosis and treatment of coronary artery vasospasm can have a determinant effect on prognosis and mortality, as appropriate treatment can lead to prolonged event-free survival. Provocative coronary vasospasm tests performed in patients with atypical cardiovascular manifestations can allow for the timely diagnosis of vasospasm and avoid critical events.

The authors aim to raise awareness of the different clinical presentations of coronary artery vasospasm and its consequences. The performance of provocative tests in selected patients should be considered to promote early diagnosis and potentially avoid major events.

## Introduction

Sudden cardiac death (SCD) is the cause of approximately 50% of cardiovascular deaths, and it can be the first manifestation of cardiac disease in up to 50% of cases. SCD is frequently caused by arrhythmic events [1-5]. Its incidence increases with age and is higher in men, possibly due to the higher incidence of cardiovascular risk factors and coronary artery disease in men [1,5]. In Europe, 10%-20% of deaths are SCD, and coronary artery disease is responsible for up to 75%-80% of SCD [1-3]. SCD may be caused by primary electrical diseases, cardiomyopathies, myocarditis, valvular heart diseases, or coronary artery disease (including acute coronary syndrome) [4,5].

The diagnostic approach to an SCD survivor includes a 12-lead electrocardiogram (ECG) to exclude electrical instability and findings suggestive of ongoing ischemia (such as ST segment changes), an echocardiogram to diagnose possible structural heart disease (including cardiomyopathy and valvular heart disease) or segmental wall abnormalities, and the exclusion of extracardiac causes of cardiac arrest (including electrolyte/metabolic imbalance). In patients with no ischemia and no noncardiac causes for cardiac arrest, repeated/reviewed ECG should be analyzed for findings suggestive of preexcitation or primary electrical disease. If ECG and echocardiogram are nondiagnostic, coronary artery angiogram or computed tomography (CT) should be considered, as well as cardiac magnetic resonance, in order to exclude coronary artery anomalies, myocardial edema and infiltration, perfusion defects, and fibrosis (suggested by late gadolinium enhancement). Exercise tests and sodium blocker tests can be used to aid the diagnosis of primary electrical disease. Coronary artery vasospasm can be suspected in patients with no other identifiable cause for SCD or in patients with typical symptoms or transient ST elevation [3].

Coronary artery vasospasm is defined as a transient total or subtotal coronary artery obstruction (>90%), associated with angina symptoms and ischemic ECG findings [6]. It was first described by Prinzmetal et al. [7] in 1959, and it is a recognized cause of myocardial infarction, life-threatening arrhythmias, atrioventricular block, and SCD [8]. Standard diagnostic tests may lack abnormalities in coronary vasospasm patients, due to the transient nature of symptoms and ischemic/arrhythmic events. Traditional cardiovascular risk factors seem to be less important for the risk of vasospasm, in contrast to other forms of coronary artery disease [2]. Smoking seems to be the most important risk factor, with a higher prevalence of vasospasm also described in patients with migraine and Asian populations. Genetic mutations may have a role in increasing susceptibility to vasospasm, including mutations in the endothelial nitric oxide synthase and other molecules involved in the modulation of vascular tone [8,9]. Associations with alcohol consumption and sympathomimetic agents (such as cocaine, adrenaline, and ergotamine) have also been described [8,10].

The gold standard for the diagnosis of coronary artery vasospasm includes the administration of a provocative stimulus (typically intracoronary acetylcholine or intravenous {IV} ergonovine) during coronary artery angiography while monitoring ECG and symptoms. These drugs cause vasoconstriction due to their action in the muscarinic and serotoninergic receptors [11]. Patients with unexplained resuscitated cardiac arrest or unexplained syncope with antecedent chest pain should be considered for provocative coronary spasm testing, but this is not common practice in many centers due to the lack of the availability of provocative tests, low clinical suspicion, or the lack of technical expertise [8,12].

## Case presentation

A 55-year-old man presented to the accident and emergency department following an out-of-hospital cardiac arrest (OHCA). He had a previous history of obesity, dyslipidemia, smoking, excessive alcohol intake, and hyperuricemia and a recent diagnosis of non-obstructive coronary artery disease and paroxysmal atrial fibrillation, diagnosed when investigating two recent episodes of syncope and thoracic pain.

A month prior to admission, he had been admitted to the hospital under continuous ECG monitoring for 14 days due to complaints of angina and an episode of syncope (with no associated trauma or lesions). Cardiovascular stratification included a normal ECG (sinus rhythm and no ST segment abnormalities) and a normal transthoracic echocardiogram with preserved biventricular function. Cardiac catheterization reported nonocclusive (30%-40%) stenosis of the left main coronary artery and the milking of the medium segment of the anterior descending coronary artery (Video 1). No arrhythmic events or thoracic pain occurred during hospitalization. He was scheduled for an exercise myocardial perfusion scintigraphy and follow-up cardiology appointment due to the suspicion of transient exercise-triggered ischemic/arrhythmic events. The patient had a new episode of syncope and thoracic pain during exercise 10 days before admission, with the documentation of paroxysmic atrial fibrillation. He was initiated on hypocoagulation and anti-arrhythmic medication (propafenone), and an insertable cardiac monitor (ICM) (Medtronic LINQ II®, Minneapolis, MN) was placed. A cardiac magnetic resonance was requested for further investigation (but not performed before the new hospital admission).

**Video 1:** Coronary angiography Left main coronary artery with 30%-40% stenosis; the milking of the mid-segment of the left anterior descending artery; right coronary artery and left circumflex artery with irregularities

**Figure 1:**
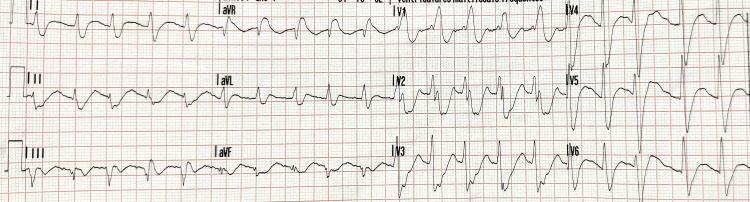
Pre-hospital post-return of spontaneous circulation electrocardiogram Sinus tachycardia; broad QRS; right bundle branch block; diffuse ST segment depression with ST elevation in aVR aVR: augmented vector right

**Figure 2:**
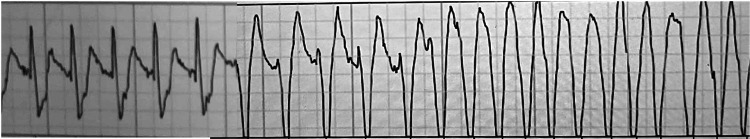
Insertable cardiac monitor review Progression from sinus tachycardia to ventricular tachycardia and ventricular fibrillation

On the day of hospital admission, the patient reported complaints of dizziness throughout the day. He did not seek medical assistance or start any medication for this complaint. At night, during physical activity, he complained of dizziness and collapsed. Cardiac arrest was promptly identified, and basic life support (BLS) was initiated. A pre-hospital advanced life support (ALS) team arrived after 20 minutes of BLS and identified ventricular fibrillation. He underwent four cycles of advanced life support (including adrenaline 1 mg twice, according to the ALS algorithm), with the initial rhythm evaluation revealing ventricular fibrillation and return of spontaneous circulation (ROSC) after eight minutes of ALS. Post-ROSC ECG revealed sinus tachycardia with broad QRS complexes and right bundle branch block, with ST elevation in augmented vector right (aVR) and diffuse ST depression (Figure 1). A review of the ICM revealed no abnormal findings during the day and an episode of sinus tachycardia preceding non-sustained ventricular tachycardia and ventricular fibrillation with a total duration of 20 minutes at the time of the collapse (Figure 2). The patient was intubated, ventilated, and transported to the hospital.

**Figure 3:**
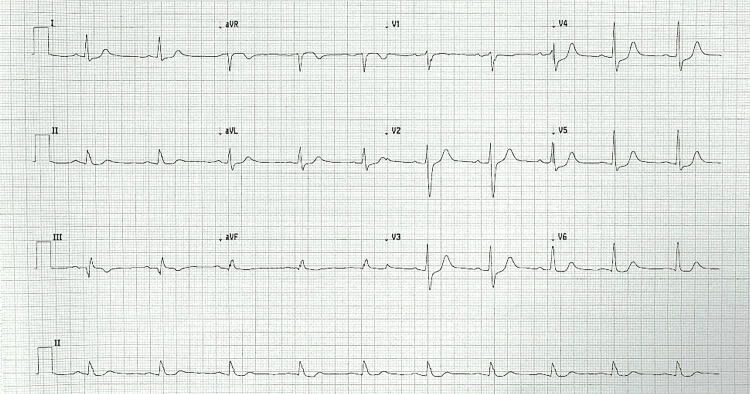
Electrocardiogram one hour post-return of spontaneous circulation

On hospital admission, he was hemodynamically stable with no vasopressor therapy, with a Glasgow Coma Scale of 3 without any sedation. The ECG recorded one hour post-ROSC showed sinus rhythm without ST segment abnormalities (Figure 3). Blood analysis excluded electrolyte imbalance or other significant abnormalities. Initial high-sensitivity troponin I was 182 ng/L. Due to hemodynamic and electrical stability and no suspicion of ongoing ischemia, no anti-arrhythmic drugs were initiated. He was admitted to the intensive care unit (ICU) for post-resuscitation care.

**Figure 4:**
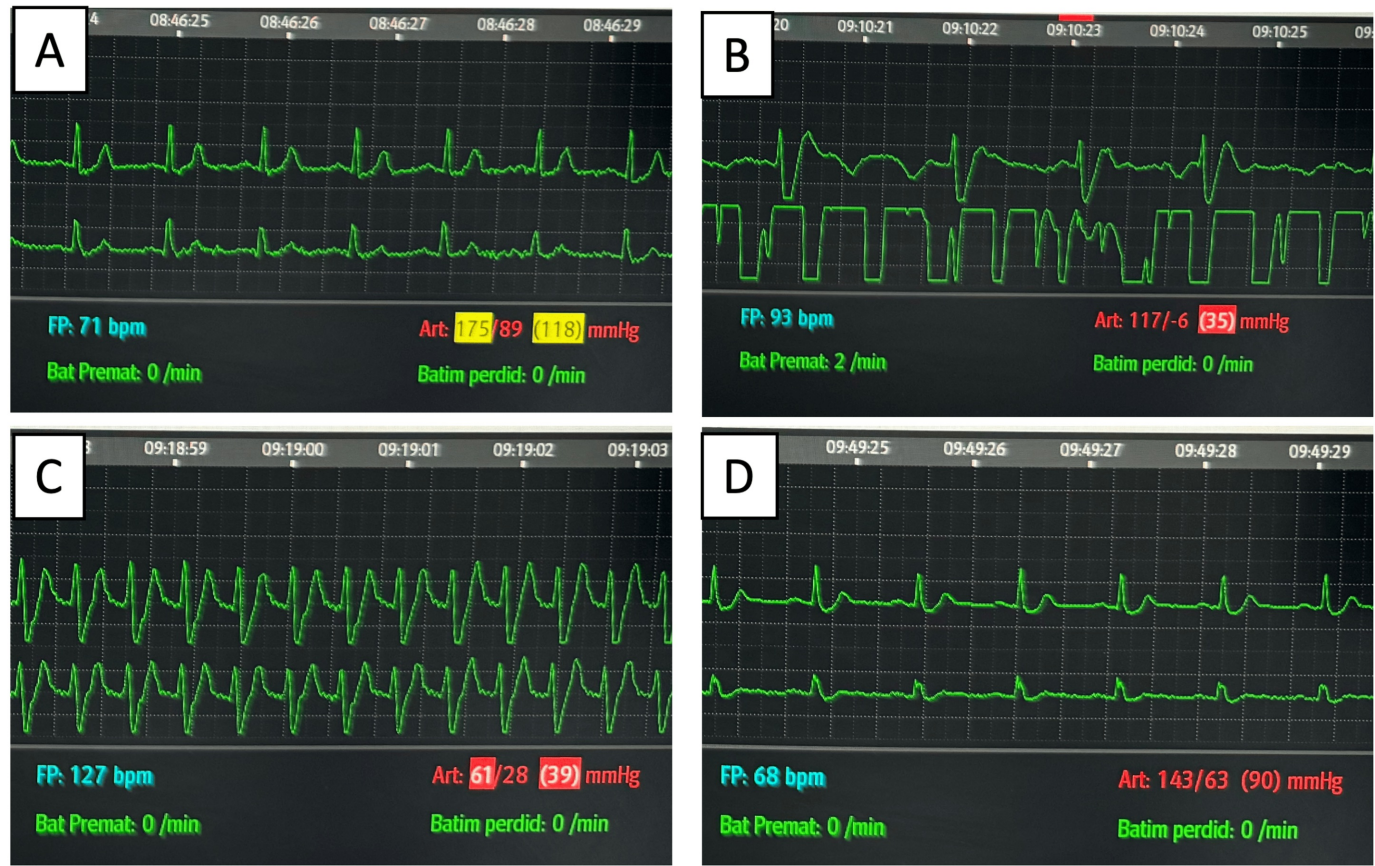
Electrocardiographic monitoring before, during, and after the first in-hospital cardiac arrest (A) Sinus rhythm; (B) atrial fibrillation with aberrant conduction; (C) ventricular fibrillation; (D) sinus rhythm (post-return of spontaneous circulation)

Nine hours after admission, a new cardiac arrest occurred, with a period of sinus tachycardia, followed by atrial fibrillation with aberrant conduction and then by ventricular tachycardia and ventricular fibrillation (Figure 4). ROSC occurred after four minutes. The post-ROSC ECG revealed no abnormalities. An echocardiogram was performed, with no segmental contractility changes, no valve abnormalities, and a preserved biventricular ejection fraction (Video 2). Maximum troponin I level was 2807 ng/L on the morning after admission, with a subsequent descending tendence. This was interpreted in the context of myocardial injury, with no temporal evolution or other findings suggestive of primary ischemic events. The patient was kept under deep sedation, and therapy with amiodarone and bisoprolol was initiated. These were chosen in accordance with current practice on the treatment of ventricular arrhythmias, with the choice of a beta-blocker motivated by the existence of sinus tachycardia preceding each of the cardiac arrests [3].

**Video 2:** Echocardiogram post-return of spontaneous circulation after the first in-hospital cardiac arrest

**Figure 5:**
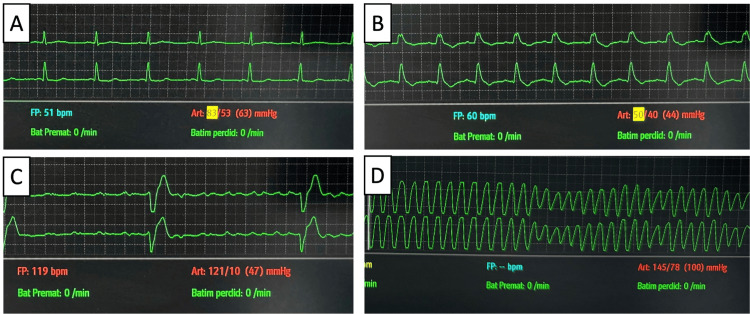
Electrocardiographic monitoring during the second in-hospital cardiac arrest (A) Sinus rhythm; (B) the broadening of QRS; (C) atrial flutter with aberrant conduction; (D) ventricular fibrillation

Twenty-six hours later, a new cardiac arrest occurred. ECG monitoring revealed the broadening of QRS following a period of raised heart rate, followed by atrial flutter with aberrant conduction and ventricular fibrillation, with ROSC after 10 minutes of advanced life support (Figure 5). Intravenous verapamil was initiated due to the recurrence of ventricular tachycardia despite amiodarone, in accordance with current guidelines [3]. A switch to metoprolol with a target heart rate of <80 beats per minute (bpm) was later made due to a shortage of IV verapamil stock.

The case was discussed with an extracorporeal life support (ECLS) reference center to assess the possibility of mechanical support in the event of new episodes of cardiac arrest. The patient was deemed non-eligible until further neurological prognostication could be performed.

**Figure 6:**
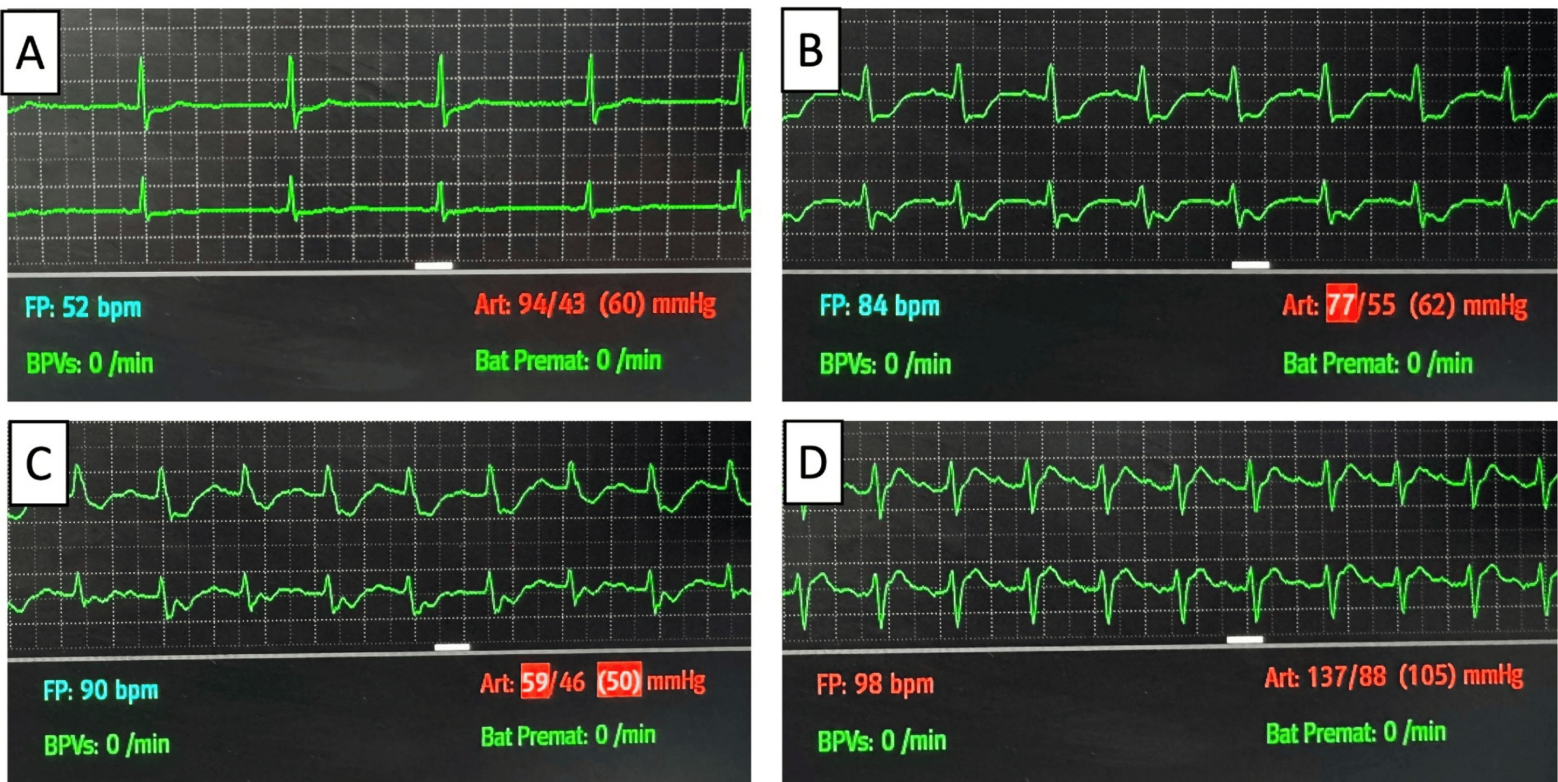
Electrocardiographic monitoring during the third in-hospital cardiac arrest (A) Sinus rhythm; (B) ST depression and QRS broadening; (C) broad QRS in pulseless electrical activity; (D) sinus rhythm

**Figure 7:**
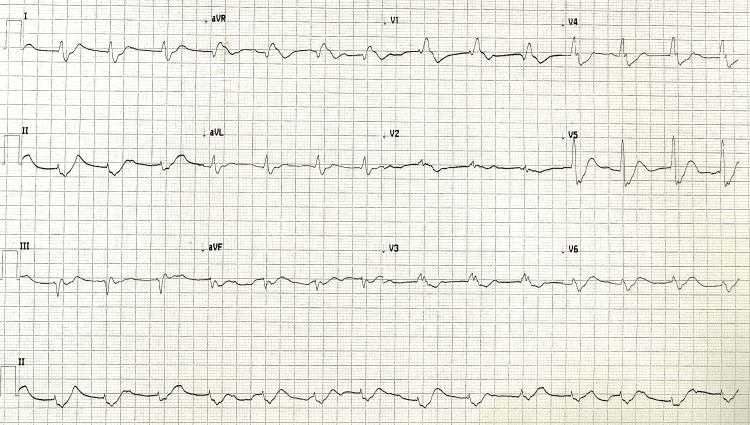
Electrocardiogram after the third in-hospital cardiac arrest ST elevation in aVR; diffuse ST depression aVR: augmented vector right

**Figure 8:**
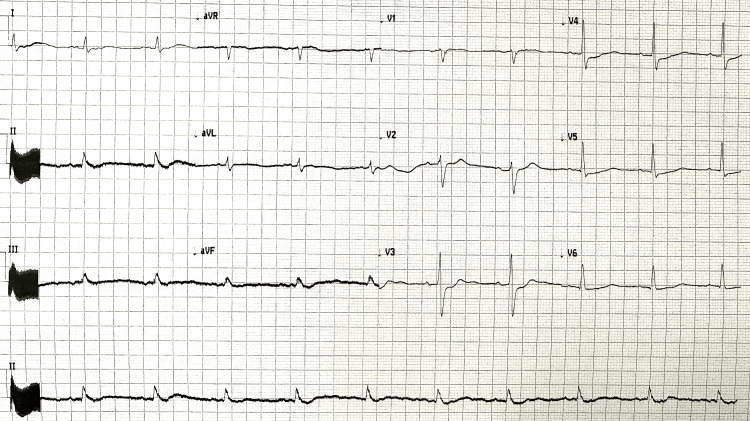
Electrocardiogram one hour post-return of spontaneous circulation

On the third day after admission (35 hours after the last cardiac arrest), ECG monitoring revealed new-onset ST depression with progressive worsening, followed by an increase in heart rate, broadened QRS complexes, and cardiac arrest in pulseless electrical activity, recovered after 15 minutes of ALS (Figure 6). Post-ROSC ECG revealed atrial fibrillation with ST elevation in aVR and diffuse ST depression (Figure 7). An echocardiogram performed immediately after ROSC revealed significant segmental motility abnormalities, with akinesis of the apex, anterior wall, and distal portion of the interventricular septum (Video 3). The patient evolved with post-cardiac arrest hemodynamic instability with the need for vasopressor support. Progressive hemodynamical improvement was observed, and the ECG and echocardiogram were repeated one hour after ROSC. The ECG revealed sinus rhythm with the resolution of the ST segment abnormalities previously observed (Figure 8). The echocardiogram revealed preserved biventricular ejection fraction and the resolution of the segmental changes previously observed.

**Video 3:** Echocardiogram after the fourth in-hospital cardiac arrest Akinesia of the apex, anterior wall, and distal half of the interventricular septum

The hypothesis of left main coronary artery vasospasm was considered, and the patient initiated anti-ischemic therapy with nitrate. The case was discussed with angiography centers, and the patient was considered not to have an indication for emergent cardiac catheterization. This was based on hemodynamic and electrical instability, the uncertain neurological prognosis, the lack of the availability of provocative tests on an emergent angiography, and the high risk of adverse events of provocative tests in the critically ill patient.

**Figure 9:**
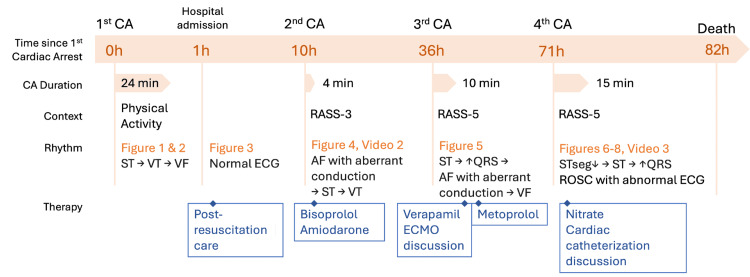
Clinical case timeline AF, atrial fibrillation; CA, cardiac arrest; ECG, electrocardiogram; ECMO, extracorporeal membrane oxygenation; RASS, Richmond Agitation-Sedation Scale; ROSC, return of spontaneous circulation; ST, sinus tachycardia; STseg, ST segment; VF, ventricular fibrillation; VT, ventricular tachycardia

During hospital admission, a thorough neurological examination was not possible due to the need for deep sedation to prevent adrenergic activation. On the second day post-admission, a motor response with flexion to pain was observed during a period of lighter sedation. A cerebral CT was performed on the fourth day post-admission (80 hours after the OHCA) for neurological prognostication, showing diffuse cerebral edema and the loss of cortico-subcortical differentiation predictive of an adverse neurological outcome. The neuron-specific enolase at 72 hours post-OHCA was 102.7 ng/mL (predictive of adverse neurological prognosis if >60 ng/mL). Due to these findings predictive of an adverse neurological prognosis, a strategy of strict symptomatic treatment was initiated, and the patient died later that day [13]. Figure 9 summarizes the timeline of events.

The patient underwent perimortem genetic testing with a comprehensive arrhythmia next-generation sequencing panel, which did not identify any pathogenic mutation. An autopsy was not performed, in accordance with the family's decision.

## Discussion

Left main coronary artery vasospasm is a very rare condition with only a few cases reported in the literature. Clinical presentations are varied and range from angina to acute coronary syndromes and malignant ventricular arrhythmias that may cause sudden cardiac death. The role of the left main coronary artery in myocardial perfusion determines that severe obstruction commonly causes malignant arrhythmias or sudden cardiac death, making the diagnosis of vasospasm particularly challenging [10,14,15]. Syncope has been reported as a manifestation of coronary vasospasm, either due to hypotension associated with low cardiac output or due to transient sinus or atrioventricular block caused by ischemia [9,16-18].

In our case, the patient had been previously admitted and studied due to complaints of angina (two episodes) and syncope (one episode). Symptoms were first attributed to atrial fibrillation with rapid ventricular response, and continuous cardiac monitoring was initiated after these episodes. The patient was referred for cardiology follow-up, and exercise testing was scheduled to exclude ischemic events as a cause of the symptoms. The lack of ECG and echocardiographic abnormalities and the findings during angiography directed the team toward the suspicion and investigation of more common causes for the patient's symptoms, and vasospasm was not considered likely at that moment. We believe that a cardiac magnetic resonance (requested but not scheduled before the OHCA) could also be included in the diagnostic approach to this case, as it could suggest alternative diagnosis, such as primary arrhythmogenic cardiomyopathies.

Even though the patient was under ECG monitoring during the episodes of cardiac arrest, the manifestations were atypical, with sinus tachycardia preceding ventricular fibrillation or atrial fibrillation with aberrant conduction preceding ventricular tachycardia and ventricular fibrillation. This contrasts with more classical arrhythmic causes of cardiac arrest, such as primary malignant ventricular arrhythmias. The lack of ST segment or echocardiographic changes (that would be typical of ischemic events) in the first events delayed the diagnosis, which often occurs in coronary vasospasm. The absence of thoracic pain preceding the cardiac arrest and the nontypical ECG and echocardiographic findings raised the suspicion of primary arrhythmic causes, and the patient was treated with anti-arrhythmic therapy and sedation to decrease adrenergic activation, according to current practice guidelines for the management of ventricular arrhythmias [3].

The suspicion of left main coronary artery vasospasm was raised after the fourth cardiac arrest, due to ECG and echocardiographic findings suggestive of left main coronary ischemia that resolved rapidly and spontaneously after the event. Unfortunately, a provocative coronary spasm test was not performed, as the hemodynamic reference centers considered that there was a lack of benefit in emergent catheterization after the normalization of ECG and echocardiographic findings. This decision was based on the lack of the availability of provocative tests on emergent procedures (considered essential for definitive diagnosis after the normalization of ECG/echocardiographic findings) and the patient's electrical and hemodynamic instability. The performance of provocative tests in critically ill patients is associated with the risk of further instability and adverse outcomes [11]. Elective testing after the acute phase could have been considered if the patient had survived the acute event.

This case highlights the different clinical presentations of coronary vasospasm. Atrioventricular block caused by transient ischemia has been described in patients with coronary vasospasm and may be a cause of syncope due to bradycardia and subsequent cerebral hypoperfusion [9,18]. The sudden severe left ventricular dysfunction can also cause cerebral hypoperfusion and the associated loss of consciousness.

Coronary artery vasospasm is an underdiagnosed cause of major cardiac events [8,12,15]. Diagnosis can be very challenging due to the transient findings and varied clinical manifestations, often in patients with normal coronary arteries. In patients with risk factors for coronary artery disease, as in our case, symptoms can be attributed to more frequent diagnoses such as coronary artery disease or atrial arrhythmias. The correct diagnosis and timely treatment of coronary artery vasospasm can have a determinant effect on prognosis and mortality, as directed treatment with calcium channel blockers can lead to prolonged event-free survival [15,19]. Provocative coronary vasospasm tests performed in patients with atypical cardiovascular manifestations can allow for the timely diagnosis of vasospasm and avoid critical events. In our patient, the hypothesis of vasospasm was not considered likely in the first clinical presentations of anginal pain and syncope, and provocative testing was not performed in the diagnostic cardiac angiography.

With this manuscript, the authors aim to raise awareness of the different clinical presentations of coronary artery vasospasm and its consequences. The routine performance of provocative tests in selected patients should be considered to promote early diagnosis and potentially avoid major events.

## Conclusions

Coronary artery vasospasm is a rare cause of major cardiovascular events. It is often under-recognized due to its transient nature and diverse clinical presentations. High clinical suspicion and the decision to perform provocative tests can have a determinant impact on the diagnosis and prognosis of these patients. Early diagnosis and directed therapy can allow for long-term event-free survival. Atypical presentations such as malignant arrhythmias can be present and make the diagnosis particularly challenging. The need for high clinical suspicion to improve outcomes in these patients makes raising awareness for this diagnosis of the utmost importance in current clinical practice.
